# An Unusual Cause of Dysphonia with Hemoptysis: A Laryngeal Live Leech 

**Published:** 2014-07

**Authors:** Mohsen Rajati, Shirin Irani, Ehsan Khadivi, Mehdi Bakhshaee

**Affiliations:** 1 *Sinus and Surgical Endoscopic Research Center, Ghaem Hospital, Faculty of Medicine, Mashhad University of Medical Sciences, Mashhad, Iran.*; 2 *Department of Otorhinolaryngology, Ghaem Hospital, Mashhad University of Medical Sciences, Mashhad, Iran.*

**Keywords:** Dysphonia, Hemoptysis, Leech

## Abstract

**Introduction:**

Foreign bodies in the upper airway are one of the most challenging otolaryngology emergencies and have various presentations depending on their physical properties and location. Leeches are blood-sucking hermaphroditic worms that vary in color, length, and shape. They usually reside in fresh-water streams and lakes. When rural untreated water is drunk, leeches may localize in the nose, pharynx, and esophagus, or rarely in the larynx.

**Case Report:**

This case is a man who was referred to our otolaryngology clinic with a complaint of hemoptysis and mild respiratory distress. The patient’s symptoms were all relieved post operatively and he was discharged on the second day following the procedure.

**Conclusion:**

Leeches should be suspected as an airway foreign body in patients with a recent history of drinking stream water.

## Introduction

Foreign bodies in the upper airway are one of the most challenging otolaryngology emergencies and have various presentations depending on their physical properties and location. Leeches are blood-sucking hermaphroditic worms that vary in color, length, and shape. They usually reside in fresh-water streams and lakes. When rural untreated water is drunk, leeches may localize in the nose, pharynx, and esophagus, or rarely in the larynx (1). The symptoms of a laryngeal leech include hemoptysis, dysphonia, stridor, choking, respiratory distress, and foreign body sensation. In this report we describe a case of a live leech located in a patient’s larynx and the technique used to remove it.

## Case Report

A 64-year-old man was referred to our otolaryngology clinic at a tertiary university hospital with a complaint of hemoptysis. He felt the sensation of a foreign body after drinking stream water eight days before. Six days after drinking the water the patient developed hemoptysis and dysphonia; he did not report any odynophagia or dysphagia but had mild respiratory distress. Indirect laryngoscopic examination revealed a dark green live leech in the anterior supraglottic region with extension toward the glottis. 

**Fig 1:**
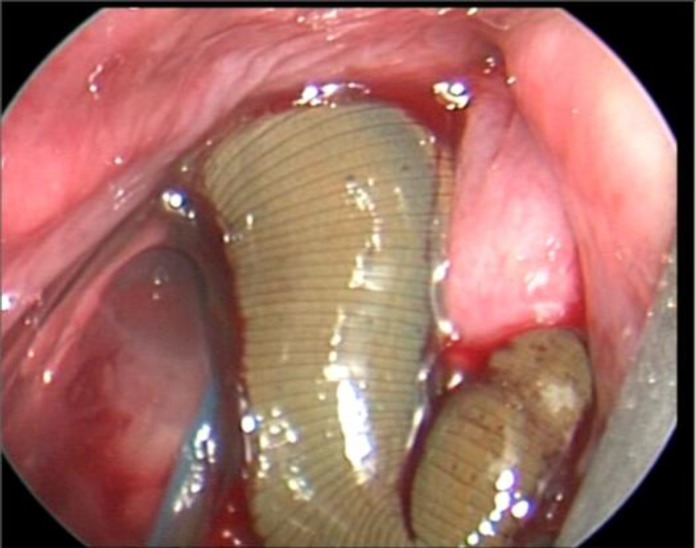
Laryngoscopic liew

**Fig 2:**
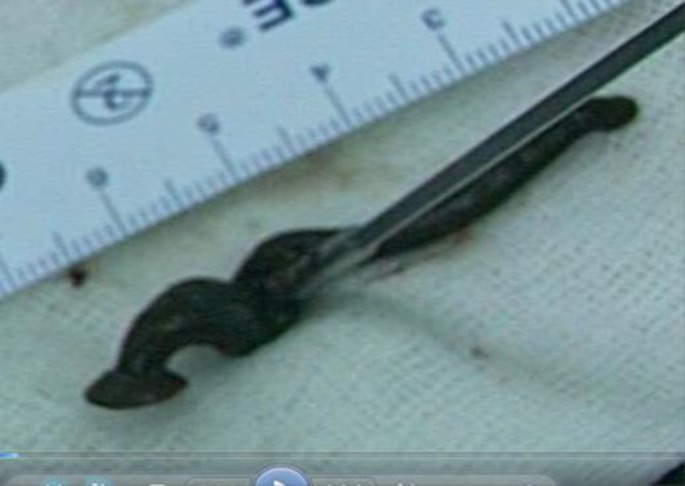
Extracted live leech to compare size

The patient was urgently admitted to the operating room. After induction of general anesthesia, a number 6 orotracheal tube was cautiously placed as far posteriorly as possible, taking care not to touch the leech. A rigid laryngoscope was then placed, pushing the epiglottis anteriorly, and exposing the endolatynx. The laryngoscope was then suspended and the larynx was examined with a 0º rigid telescope. Located on the anterior supraglottic region was a live leech with its sucking head stuck to the anterior commissure just below the vocal cords (Fig. 1). As shown in the attached video, the leech was gently grasped with foreign body forceps and we patiently waited until the leech loosened its grasp so it could be removed undamaged and intact with its full length of more than 5.5 Cm (Fig 2). The patient’s symptoms were all relieved post operatively and he was discharged on the second day following the procedure. The Mashhad University of medical Sciences institutional review board has approved this report. 

## Discussion

Live foreign bodies in the upper aerodigestive system are rare. There are only a few reports of live leeches, ascaris, and fish in the larynx (2). Leeches can attach to the mucosa of the entire upper aerodigestive tract but a leech in the larynx is very rare (3). Interestingly, the first report of leeches lodged in the upper airway system goes back to the tenth century and was recorded by the Persian physician Rasis^4^. Rahimi-Rad and colleagues also reported a 73-year-old man with a complaint of intermittent hemoptysis, dysphagia, dyspnea, and stridor who had a history of recent contact with stream water. A fiberoptic laryngoscopy revealed a glottic live leech which was removed following application of lidocaine (5).

Direct laryngoscopy under general anesthesia is the best approach for removal of a leech from the aerodigestive tract. As a leech attaches strongly with its sucking head, removal should be done so gentle and cautiously. How the leech is grasped is also important because it is slippery and could rupture easily; thus, using forceps with blunt jaws is recommended. Great care should also be taken to entirely remove all parts of the body. Rupture of the leech with parts of the head remaining could result in continued bleeding because the suckers contain hirudin, which is an anticoagulant enzyme. Some experts advise using lidocaine in tough cases as it causes relaxation of the head suckers.

The presence of a live leech in the respiratory tract should be suspected in patients with complaints of hemoptysis, hoarseness, and respiratory distress of unknown origin who have a history of contact with untreated water. The symptoms may be misdiagnosed as laryngitis, asthma, infections such as tuberculosis, or even malignancies (4).

## Conclusion

Despite the rarity of the occurrence, leeches should be suspected as an airway foreign body in patients with a recent history of drinking stream water. Indirect laryngoscopy can be used to make a definite diagnosis in the case of a leech in the larynx and rigid laryngoscopy is the procedure of choice to remove them.
